# Reliability of fast T1 VIBE Dixon MRI for femoral version measurement in FAI patients: A comparative study with CT

**DOI:** 10.1016/j.ejro.2026.100745

**Published:** 2026-04-13

**Authors:** Till D. Lerch, Tilman Kaim, Malin Kristin Meier, Adam Boschung, Simon D. Steppacher, Moritz Tannast, Florian Schmaranzer

**Affiliations:** a Department of Diagnostic, Interventional and Paediatric Radiology, University of Bern, Inselspital, Bern University Hospital, Bern, Switzerland; b Department of Orthopaedic Surgery and Traumatology, Inselspital, Bern University Hospital, University of Bern, Bern, Switzerland; c Department of Radiology, Faculty of Medicine, Balgrist University Hospital, University of Zurich, Switzerland

**Keywords:** Hip, MRI, Femoral version, CT, Dixon based images

## Abstract

**Purpose:**

Patients with femoroacetabular impingement (FAI) can present with abnormal femoral version(FV). Discrepancies were reported for FV Measurement comparing MRI and CT. We assessed difference of FV measurement on MRI and on matched CT.

**Methods:**

A retrospective IRB-approved comparative study involving 100 hips of symptomatic FAI patients was performed. All patients(100 hips) had hip pain(mean age 28 ± 10 years) and underwent pelvic CT scan and MRI of the same hip. Routine unilateral, multiplanar MRI of the hip was acquired for chondrolabral lesions. Of them, 52 hips(46 patients) underwent hip MRI with standard T1 images, while 48 hips underwent hip MRI with an additional fast MRI with Dixon based images(bilateral T1 Vibe Dixon based images of pelvis and knee) to measure FV. Two readers independently measured FV(Murphy method) on CT and MRI.

**Results:**

Difference of FV decreased significantly(p < 0.001) between CT and MRI with standard images from 4.9 ± 4.5° for Reader 1(7.3 ± 6.7° for Reader 2) to −0.05 ± 1.5°(r = 0.993, p < 0.001)for Reader 1 (0.73 ± 2.95°, r = 0.975, p < 0.001, for Reader 2) with fast Dixon based images.

Difference of FV of CT between two readers was 1.9 ± 3.2°(r = 0.969, p < 0.001) and was not significantly different compared to fast MRI with Dixon based images (1.1 ± 4.4°,r = 0.943, p < 0.001).

Number of hips with Difference of FV> 5° between CT and MRI were significantly(p < 0.001) reduced from 35 hips (67%,with standard images) to 2 hips (4%, Dixon based images) for reader 1.

**Conclusion:**

We changed our clinical practice and use fast MRI with T1 Dixon-based images for FV measurement. This decreased measurement errors in FV and misdiagnosis of FV and could potentially reduce CT scans.

## Highlights

• Hip MRI with fast Dixon based MRI sequence to measure FV was evaluated.
• Difference of femoral version (FV) between CT and MRI decreased with fast Dixon based MR images compared to standard MR images.
• Measurement errors in FV decreased, potentially leading to less misdiagnosis of FV.
• MRI with fast Dixon based MR images can be used in clinical routine for FV measurement.

## 1 Introduction

Abnormal femoral version (FV) was associated with extraarticular hip impingement [1] in young patients with femoroacetabular impingement (FAI). FAI is a known cause for hip pain in young patients [2] and there is recent evidence that abnormal FV outweighs or compensates the effect of cam-impingement on hip internal rotation [3]. FV affects the impingement-free range of motion of the hip joint and especially internal and external rotation [4] as well as in and out toeing of the foot [5]. A recent study showed that patients with decreased FV are at risk for an anterior intra- and extraarticular subspine FAI [6]. On the other hand, increased FV was associated with posterior intra- and extra-articular ischiofemoral impingement conflict [7]. Abnormalities of FV were found in up to 17% of symptomatic patients with hip pain evaluated for hip preservation surgery [8].

Correct quantification of FV is essential for diagnosis of abnormal FV and for severe torsional abnormalities [9]. However, variations in measurement methods and in acquisition technique were subject of controversy [10], [11] for MRI-based measurement of FV. In the literature, different measurement methods [12] and different modalities (CT, MRI or Ultrasound) were described [13], [14]. More specifically, previous studies reported relevant discrepancies between FV measurement with CT and with MRI. The fast Dixon-based images are different and have advantages (such as thinner slice capability) to better recognize anatomical landmarks (e.g. femoral head center) compared to standard images. Therefore, we assessed if FV measurement can be done reliably using fast MRI with Dixon based images in clinical routine compared to standard images.

We compared MRI- and CT-based measurements of FV to assess the reliability in terms of mean difference and correlation and the percentage of hips with a mean difference > 5°.

## 2 Patients and methods

A retrospective institutional review board approved comparative radiologic study involving a total of 100 hips was performed. All patients (100 hips) had symptomatic FAI (mean age of 28 ± 9 years, Table 1). All patients underwent pelvic CT scan and MRI of the same hip joint. Mean interval time between CT and MRI was 20 days. Of them, 52 hips (46 patients) underwent standard MRI before 2018, while 48 hips underwent fast MRI with additional Dixon based images (since 2018). The allocation to the two groups was time-dependent

**Table 1:** Demographic and radiographic description of the study groups is shown.

**Parameters**	**Total study group**	**MRI with standard images**	**MRI with Dixon images**
Hips (Patients)	100 (81)	52 (46)	48 (35)
Age (years)	28 ± 9 (17 – 51)	28 ± 9 (17 – 51)	28 ± 10 (18 – 51)
Sex (% male)	40	42	38
Side (% right)	55	56	54
Height (cm)	171 ± 10 (150 – 197)	167 ± 10 (155 – 197)	168 ± 11 (150 – 186)
Weight (kg)	70 ± 18 (46 – 118)	75 ± 21 (47 – 118)	65 ± 12 (46 – 83)
BMI (kg/m^2^)	24 ± 4 (16 – 38)	25 ± 5 (16 – 38)	23 ± 3 (20 – 29)
LCE angle (°)	30 ± 8 (8 – 56)	31 ± 9 (8 – 56)	30 ± 8 (8 – 43)
Neck-shaft angle (°)	133 ± 7 (119 – 157)	131 ± 7 (119 – 148)	134 ± 7 (124 – 157)
Alpha angle (°)	57 ± 13 (35 – 98)	52 ± 12 (35 – 84)	60 ± 13 (35 – 98)
Surgical treatment (% of hips)	38	44	31

LCE = lateral center edge; BMI = Body mass index;

and based only on chronological criteria, if the MRI was performed in 2018 the patient was part of the second group (patient selection supplementary Fig. 1). In addition to the routine unilateral, multiplanar protocol for chondrolabral lesions, bilateral Dixon based images (3D T1 VIBE Dixon of the pelvis and of the knee) were acquired for these 48 hips to measure FV (VIBE= Volume interpolated breath hold examination).

### 2.1 CT protocol

Three-dimensional CT was performed with 0.6 mm slice thickness (Somatom Definition Flash, Siemens Medical Solutions, Erlangen, Germany). The scanned volume included the entire pelvis and the distal femoral condyles using the following parameters: voltage 100 kVp; intensity 160 mAs; pitch 0.8; field of view 39 cm; voxel size 1 mm3, reconstruction kernels I31f and I70f. Patients were positioned supine, with the both feet taped together resulting in 10°–15° of internal rotation in the hip joint.

### 2.2 MRI protocol

**Fig. 1:**
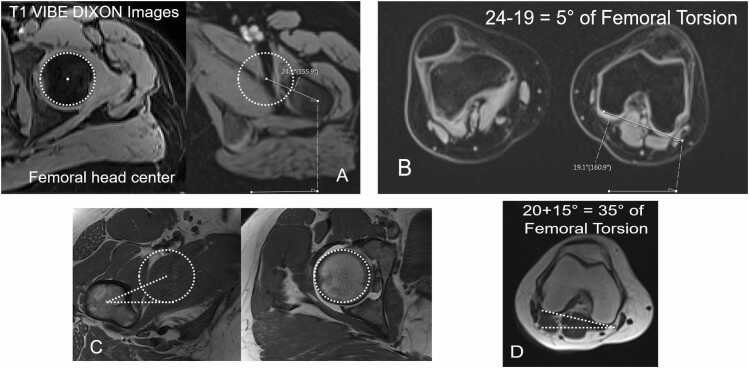
**A-D** Dixon based images (A and B) and standard T1 images (C and D) for FV measurement are shown.

We used a routine protocol for all patients for MR arthrography on 3 T scanner (Siemens Medical Solutions, Erlangen, Germany) with large flexible surface coils and multiplanar PD-w images in coronal, sagittal, axial and radial orientation. In addition, we implemented a bilateral 3D T1 VIBE Dixon sequence of the pelvis (Fig. 1) and of the knee since January 2018. The FOV of the bilateral 3D sequence of covered the entire pelvis from the anterior superior iliac spine to the level below the lesser trochanter . The FOV of the bilateral distal femur covered the entire knee joint. Acquisition time (AT) for the multiplanar standard protocol including localizers was 30 minutes. All patients were examined consecutively (2016–2019). All patients were positioned supine, with the both feet taped together. 52 hips (46 patients) underwent hip MRI with standard turbo spin echo T1 sequences (before 2018, Fig. 1). Image acquisition time was more than 3 minutes for the T1 images of the hip joint.

For the remaining patients (48 hips, since 2018) the MRI was performed with 3D T1 VIBE Dixon (Fig. 1) and included the entire pelvis (TR/TE1/TE2, 3.94/1.27/2.5ms, flip angle 9°, slice thickness of 1 mm, 312 ×400 mm field of view, a matrix size of 175 ×320 mm, anisotropic voxel size of 1.2 ×1.2x1mm, image acquisition time of 32 seconds for 192 slices). A second 3D T1 VIBE Dixon (same parameters) for the bilateral knee were acquired. In summary, image acquisition time was reduced with the T1 VIBE Dixon images.

### 2.3 Measurement of femoral version (Murphy method)

Two readers independently measured FV on both CT and MRI scans on two separate sessions using the Murphy method [15]. Both readers (blinded) that performed measurements of FV had 5 years of experience in musculoskeletal imaging. All measurements were performed on axial images without reformatting. No summation images were used. This method defined the most proximal reference on the femoral head’s center. The Murphy method uses the so-called centroid as a second proximal landmark, this is located at the center of the femoral shaft superior to the lesser trochanter at the base of the femoral neck (Figure 4). Other described methods differ regarding the definition of the second reference point to determine the proximal reference line [16]. The method described by Murphy showed better reproducibility (variance of 0.4° and a standard deviation of 0.6°) compared to one single transverse CT section through the femoral neck [17]. A line connecting the femoral condyles was used for definition of the distal reference axis. Previous investigations reported good intraobserver reliability (ICC of 0.96–0.99) and interobserver reliability (ICC of 0.98) [18]for measurement of FV using the Murphy method [16].

Difference of mean FV between CT and MRI was calculated for both readers. Then the difference of CT-based measurements of FV was compared between two readers (Supplementary Fig. 2). The number of hips with difference of FV> 5° between CT and MRI were assessed. In addition, whether the difference in FV increases with increasing FV was evaluated.

### 2.4 Statistical analysis

Normal distribution was confirmed with the Kolmogorov-Smirnov test. Because continuous parameters (FV) were normally distributed, we only used parametric tests. Pairwise comparison was performed with the independent student’s *t*-test. Correlation between FV measurement of CT and MRI was assessed using Pearson correlation coefficient (data were normally distributed). ICC (intraclass correlation coefficient) was calculated at two timepoints (reproducibility) and between two readers. Agreement was assessed between imaging modalities. Agreement was assessed with Bland-Altman analysis and 95% limits of agreement including Pearson correlation coefficients (R^2^). Bland-Altman analysis was performed for both readers.

## 3 Results

**Fig. 2:**
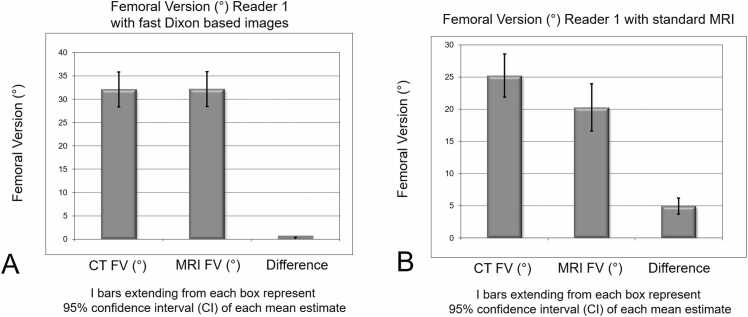
**A and B** Bar chart shows FV measurement for CT and MRI and corresponding mean difference (MD) between CT and MRI with Dixon based images (A) and MRI with standard images (B).

(1) Mean difference of FV decreased significantly (p < 0.001) between CT and MRI with standard images from 4.9 ± 4.5° for Reader 1 (7.3 ± 6.7° for Reader 2) to −0.05 ± 1.5° (Fig. 2) for Reader 1 (0.73 ± 2.95°, for Reader 2) for fast MRI with Dixon based images (Table 2).

**Table 2:** **(A)** Agreement between imaging modalities for FV measurement using CT and MRI. Accuracy in terms of mean difference. CT based measurement of FV served as gold standard.

**Parameters**	**CT**	**MRI with standard images**	**CT**	**MRI with Dixon images**
**Time period**	**2016–2017**		**2018–2020**	
FV (°) reader 1	25 ± 12 (4 – 54)	20 ± 13 (-4 – 52)	32 ± 13 (7 – 65)	32 ± 13 (6 – 66)
Mean difference (°)		4.9 ± 4.5 (-10 – 7)		-0.05 ± 1.5 (-3.0–2.9)
FV (°) reader 2	26 ± 13 (8 – 63)	19 ± 13 (-5 – 43)	34 ± 13 (8 – 67)	33 ± 13 (3 – 65)
Mean difference (°)		7.3 ± 6.7 (-21 – 12)		0.73 ± 2.95 (-6−10)
FV= femoral version; mean difference= difference compared to matched CT measurement of FV; Values are expressed as mean ± SD and range in parenthesis unless otherwise indicated.				

(B) Agreement (intrarater reliability) between imaging modalities (reader 1) is shown.								
**Method**	ICC	95% CI	R^2^	p-value	MD ± SD_D_	95% CI	Range	LOA
**MRI with standard images**	0.94	0.92,0.97	0.94	p < 0.001	4.9 ± 4.5	4.5–5.32	-10 – 7	3.7–8.3
**MRI with Dixon images**	0.97	0.95,0.99	0.993	p < 0.001	-0.05 ± 1.5	-0.7–0.6	-3.0–2.9	-3.05–2.95

**Fig. 3:**
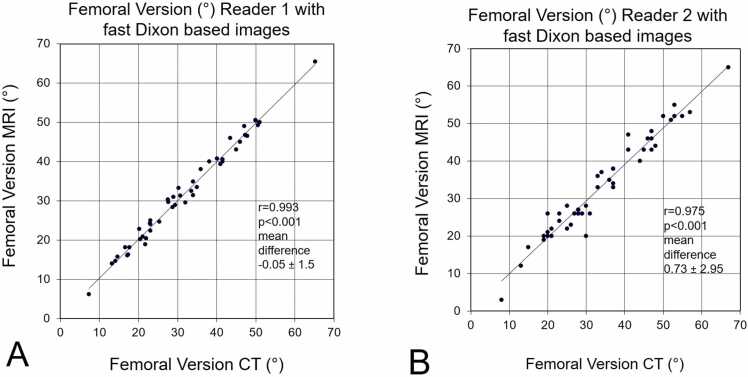
**A and B** Comparison of CT based and MRI based measurement of FV for reader 1 (A) and reader 2(B). MRI was performed with fast Dixon based images.
(2) Mean difference of CT-based measurements of FV between two readers was 1.9°±3.2 (-6–8, Table 3B) and correlation was r = 0.969 (p < 0.001). Mean difference of MRI-based measurements (with Dixon based images) of FV between two readers was 1.1°±4.4 (-10–10, Table 3B) and correlation was r = 0.943 (p < 0.001, Figure 4).

ICC = intraclass correlation coefficient, R^2^ = Pearson correlation coefficient (p < 0.01), MD = mean difference, SD_D_ = standard deviation of the difference, CI = confidence interval, LOA = limits of agreement
  Mean difference and Correlation of FV between MRI with Dixon based images and CT-based measurements was −0.05 ± 1.5° (-3.0–2.9) and r = 0.993 (p < 0.001) for reader 1 and was 0.73 ± 2.95° (-6−10) and r = 0.975 (p < 0.001) for reader 2 (Fig. 3).

**Table 3:** (A) ICC FV measurement comparing measurements at two timepoints (reproducibility) and between two readers (reliability) using CT and MRI.

**Parameters**	**CT**	**MRI with standard images**
Reproducibility (ICC reader 1)	0.958 (0.928 – 0.976)	0.93 (0.91–0.97)
Reproducibility (ICC reader 2)	0.962 (0.934 – 0.978)	0.94 (0.92–0.98)
Reliability (ICC reader 1 vs reader 2)	0.964 (0.939 – 0.979)	0.96 (0.94–0.97)

**(B)** mean difference compared between two readers (interobserver variation) using CT and MRI.				
Parameters	CT	MRI with standard images	CT	MRI with Dixon images
**Time period**	**2016–2017**		**2018–2020**	
Reliability Mean difference reader 1 vs reader 2	1.75 ± 4.1 (-9−8)	1.6 ± 4.5 (-8−19)	1.9 ± 3.2 (-6–8)	1.1 ± 4.4 (-10–10)

FV= femoral version
(3) Number of hips with difference of FV> 5° between CT and MRI were significantly(p < 0.001) reduced from 34 hips (65%, with standard images) to 9 hips (19%, with Dixon based images) for reader 2 and from 35 hips (67%, with standard images) to 2 hips (4%, with Dixon based images for reader 1.

Most differences between CT and MRI were within the corresponding ICC variation for both readers (100% for reader 1, 71% for reader 2) as shown in Bland Altman analysis (Supplementary Fig. 3).

The difference in FV between measurement modality did not increase in hips with excessive FV (correlation between MD and FV was r = 0.018 for reader 1, r = 0.025 for reader 2)

## 4 Discussion

Correct measurement of FV is crucial for diagnosis of abnormalities of FV. In addition, FV measurement is important for planning hip preservation surgery (e.g. to plan the amount of correction). However, different measurement methods and different modalities (MRI, CT and Ultrasound) were described for measurement of FV [13], [14]. In addition, previous studies reported discrepancies between CT based and MRI based measurement of FV. Thus, we compared MRI and CT to measure FV and evaluated fast MRI with Dixon based images.

Despite the increasing availability of MRI scanners and rapid techniques for assessment of FV, a controversy concerning the accuracy of FV measurement remained. Orthopedic surgeons often rely on CT for measurement of FV despite the considerable radiation exposure of CT scans. CT scans carry a lifetime risk of malignancy, especially for children and adolescents [19]. A good interrater and intrarater reliability between MRI and CT was reported for measurement of FV for adolescent patients with hip disorders [20]. They used axial images and reported reliable measurements of FV. Previously, Sutter et al. reported in 2015 [21] a difference of 3.5° between axial and axial oblique measurements of FV, with higher values of the axial method and a high interobserver agreement. More recently, differences between MRI image sequences with different image orientation have been investigated in another cohort of symptomatic patients with hip pain due to FAI [22]. They reported, that FV measurement on axial oblique sequences is more reliable than with axial MRI images [22]. Axial oblique sequences demonstrated greater measurement consistency at multiple timepoints [22].

Before the introduction of CT scans, measurements of FV were performed on radiographs or biplane radiographs. The Dunn Rippstein method is one of these historic methods described in the literature. But according to a systematic review, CT and MRI had the highest accuracy for FV measurement while limited accuracy was reported for biplane radiographs [13].

Comparing differences of FV measurements between CT and MRI, a higher correlation and lower mean difference was found in the current study compared to older studies, one study reported a correlation of 0.8 and a mean difference of 8.9° [10] while another study with a mean difference of 6–11° [11] reported a high interobserver agreement. More recently, relevant discrepancies between MR- and CT-Based FV measurements were reported for 54 patients with FAI despite strong correlations [23]. They reported that MR-based measurements were smaller than CT-based measurements, their mean differences ranged from 4.5° to 10° [23]. Another study evaluated 58 patients and reported a difference of 0.4° between MR- and CT-Based FV measurements [9] and considered MRI sufficient for assessment of FV for FAI patients. Other studies investigated FV measurements using cadaveric femurs [24], [25] and reported relevant differences between measurement methods [25]. Several studies [16], [24], [25], [26] reported differences between measurement methods for FV (e.g. Murphy method or Reikeras method). These measurement methods differed between the used proximal landmarks on axial images. Other studies compared the use of axial images and axial oblique images [21].

The current study is one of the few studies comparing CT and MRI for FV measurements using two types of MRI images for FV measurement. We found considerable differences between standard T1 images and Dixon based images. The reduced measurement errors with fast Dixon-based MRI images could be due to several reasons, such as the better contrast and better delineation of osseous structures as well as the reduced image acquisition time. The used Dixon-based MRI images had an acquisition time of less than one minute (32 seconds). Another advantage of these images is the better recognition of osseous structures, especially on the Water-based Dixon images (Fig. 1). In addition, we observed that differences between measurement methods do not increase with increasing FV. Therefore, we believe that the used method is robust.

Another strength of the current study is that no patient had previous hip operations (e.g. no previous hip arthroscopy or femoral derotation osteotomy) that could influence FV measurement. This is different compared to previous studies. One of the previous studies reported, that measuring the FV using a different method (e.g. Reikeras method) could underestimate FV. Therefore, quantifying FV was based on a measurement technique that uses the center of the femoral head (the femoral rotation’s center). This is important for patients treated with femoral derotation osteotomy [27]. Another strength of the current study is that the used T1 VIBE Dixon images reduced image acquisition time and bilateral images offer the possibility to compare FV of both sides of the patient (to calculate side-to side difference).

### 4.1 Limitations

This study has limitations. Because of the study’s retrospective design, we cannot rule out a small selection bias since the decision to perform preoperative MRI was not standardized and this decision was also dependent on patients’ factors and the practices of the different surgeons. However, the two patient cohorts are patients evaluated consecutively for hip pain over a 4year period and patient selection was only based on chronological criteria. Second we did not evaluate 3D measurement methods [12]. This is also an advantage because the used measurement method of FV and the Dixon based images in the current study are used in clinical routine and this is usually not the case for 3D measurement methods. The higher costs of MRI compared to CT and radiographs could limit generalizability and worldwide application theoretically. In addition, we evaluated young patients without end stage osteoarthritis.

## 5 Conclusion

The correct amount of FV is crucial for diagnosis of abnormalities of FV. MRI-based measurement of FV is as accurate and reliable as CT-based measurements when using fast MRI with Dixon based images for FAI patients. Usage of Dixon based images decreased measurement errors in FV, leading to less misdiagnosis of FV. We changed our clinical practice and use fast MRI with Dixon based images for preoperative measurement of FV. This could reduce CT scans and radiation dose for typically young FAI patients of childbearing age.

## Ethics

Each author certifies that his or her institution approved the human protocol for this investigation, that all investigations were conducted in conformity with ethical principles of research, and that informed consent for participation in the study was obtained.

## Ethical statement

The work involves the use of human subjects and was carried out in accordance with the World Medical Association Declaration of Helsinki.

The manuscript follows the International Committee of Medical Journal Editors (ICMJE) recommendations for the conduct, reporting, editing and publication of scholarly work in medical journals.

All procedures were performed in compliance with relevant laws and institutional guidelines and was approved by the appropriate institutional committee(s).

the date and reference number of the ethical approval(s) obtained: 13th February 2018.

Project 2018–00078

The privacy rights of human subjects were observed and informed consent was obtained for experimentation with human subjects.

## Funding statement

This research did not receive any specific grant from funding agencies in the public, commercial, or not-for-profit sectors.

## CRediT authorship contribution statement

**Florian Schmaranzer:** Writing – original draft, Validation, Methodology, Investigation, Conceptualization. **Lerch Till:** Writing – review & editing, Writing – original draft, Visualization, Supervision, Resources, Project administration, Methodology, Investigation, Funding acquisition, Conceptualization. **Tilman Kaim:** Visualization, Investigation, Data curation. **Steppacher Simon:** Supervision, Conceptualization. **Moritz Tannast:** Supervision, Conceptualization. **Adam Boschung:** Formal analysis, Data curation. **Meier Malin:** Formal analysis, Data curation.

## Declaration of Competing Interest

The authors declare that they have no known competing financial interests or personal relationships that could have appeared to influence the work reported in this paper.

## Appendix A Supplementary material

Suppl Figure 1 Flowchart of the two patient cohorts is shown

Suppl Figure 2 **A and B** Comparison of and MRI based (A) inter-observer measurements and CT based measurement (B) of FV between two readers. MRI was performed with fast Dixon based images

Suppl Figure 3 **A-B** Bland Altman analysis showing the difference between CT based and MRI based measurement of FV for reader 1 (A) and reader 2 (B). MRI was performed with fast Dixon based images

## Footnotes

Appendix A Supplementary data associated with this article can be found in the online version at doi:10.1016/j.ejro.2026.100745.
