# Supplementary figures and images for: Reliability of fast T1 VIBE Dixon MRI for femoral version measurement in FAI patients: A comparative study with CT
Source: Eur J Radiol Open. 2026 Apr 13;16:100745. doi: 10.1016/j.ejro.2026.100745 (PMC13092753; doi:10.1016/j.ejro.2026.100745)

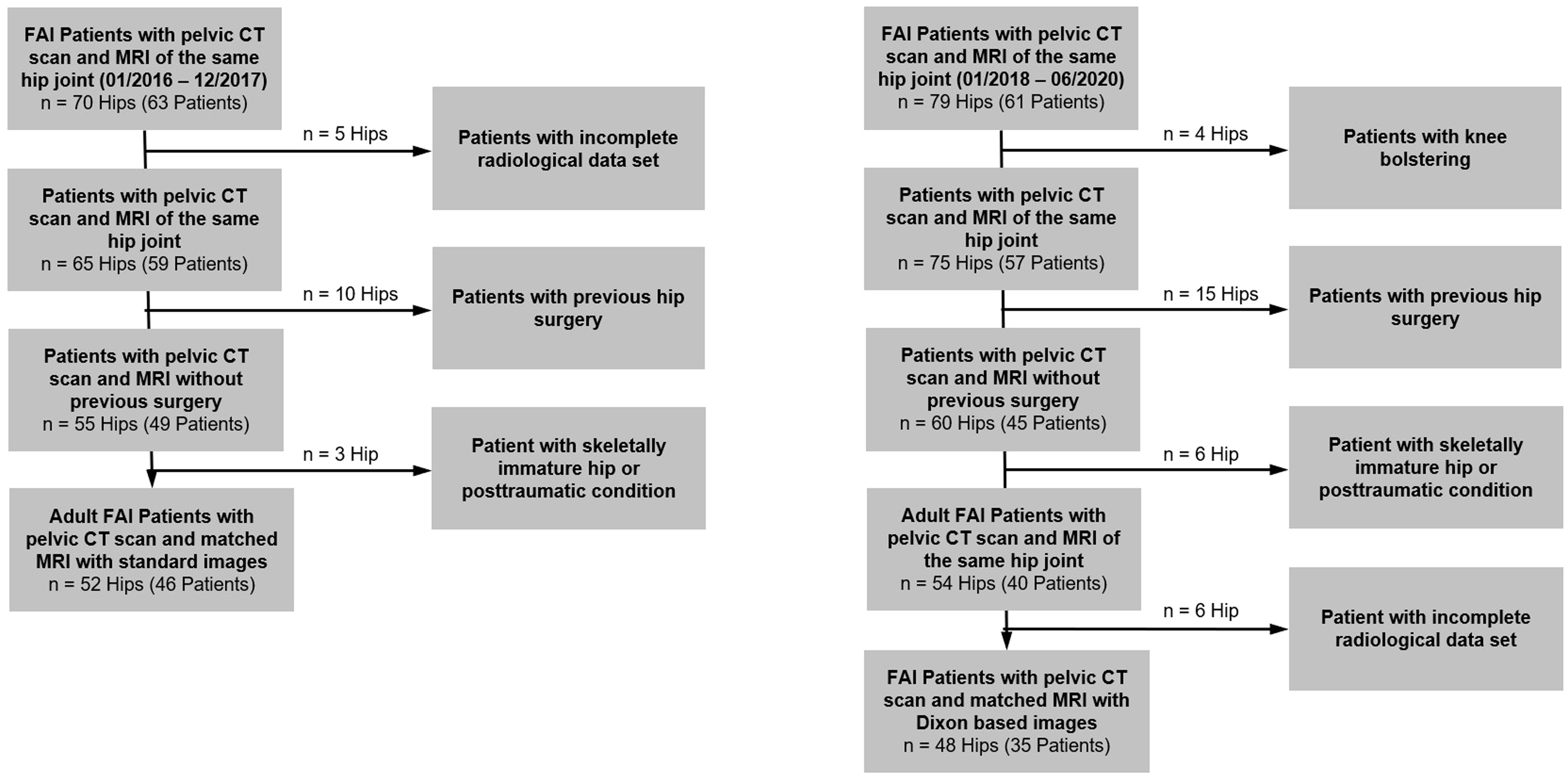

Supplement: Suppl Figure 1 — Flowchart of the two patient cohorts is shown [file mmc1.jpg]

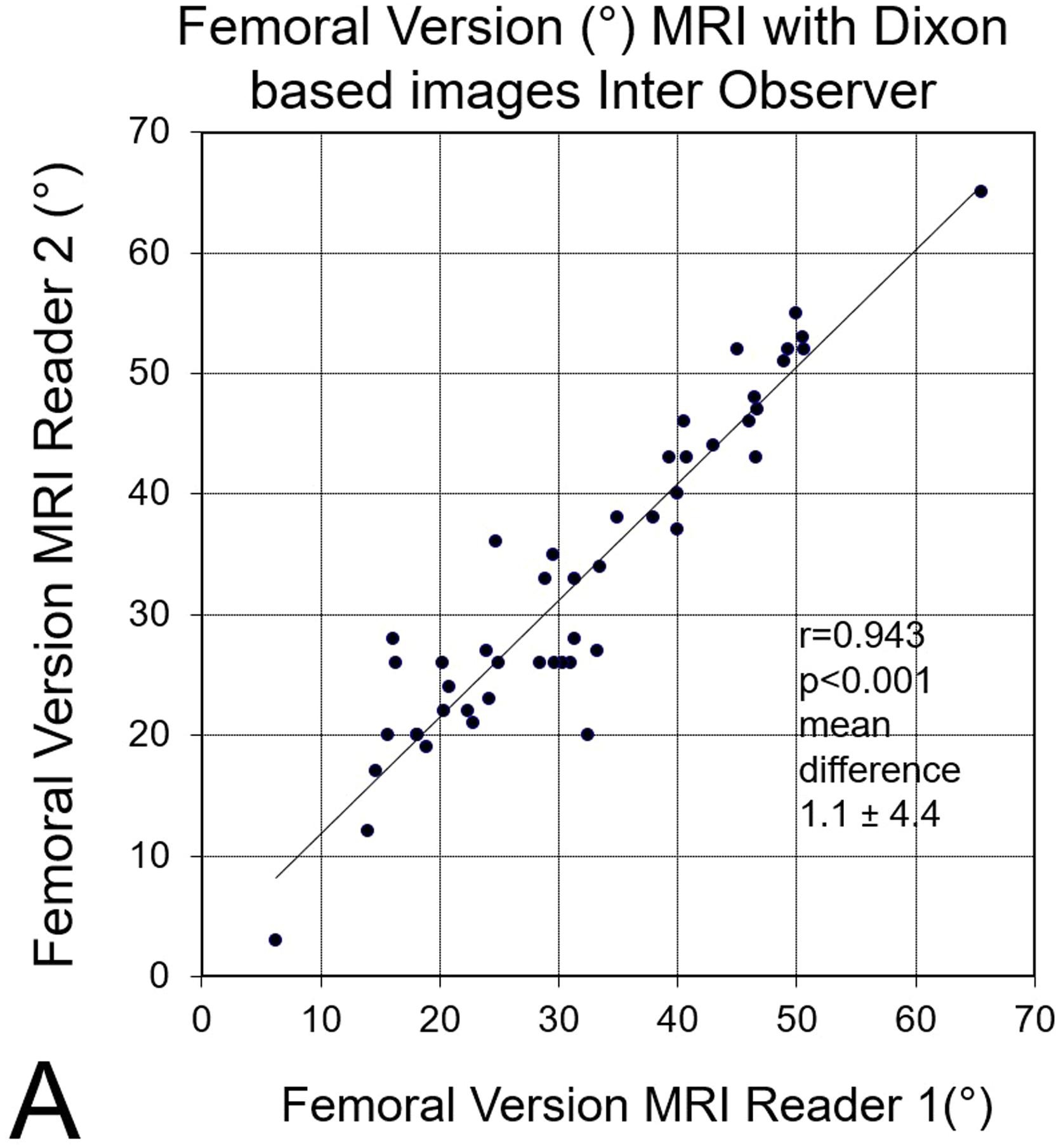

Supplement: Suppl Figure 2 — A and B Comparison of and MRI based (A) inter-observer measurements and CT based measurement (B) of FV between two readers. MRI was performed with fast Dixon based images [file mmc2.jpg]

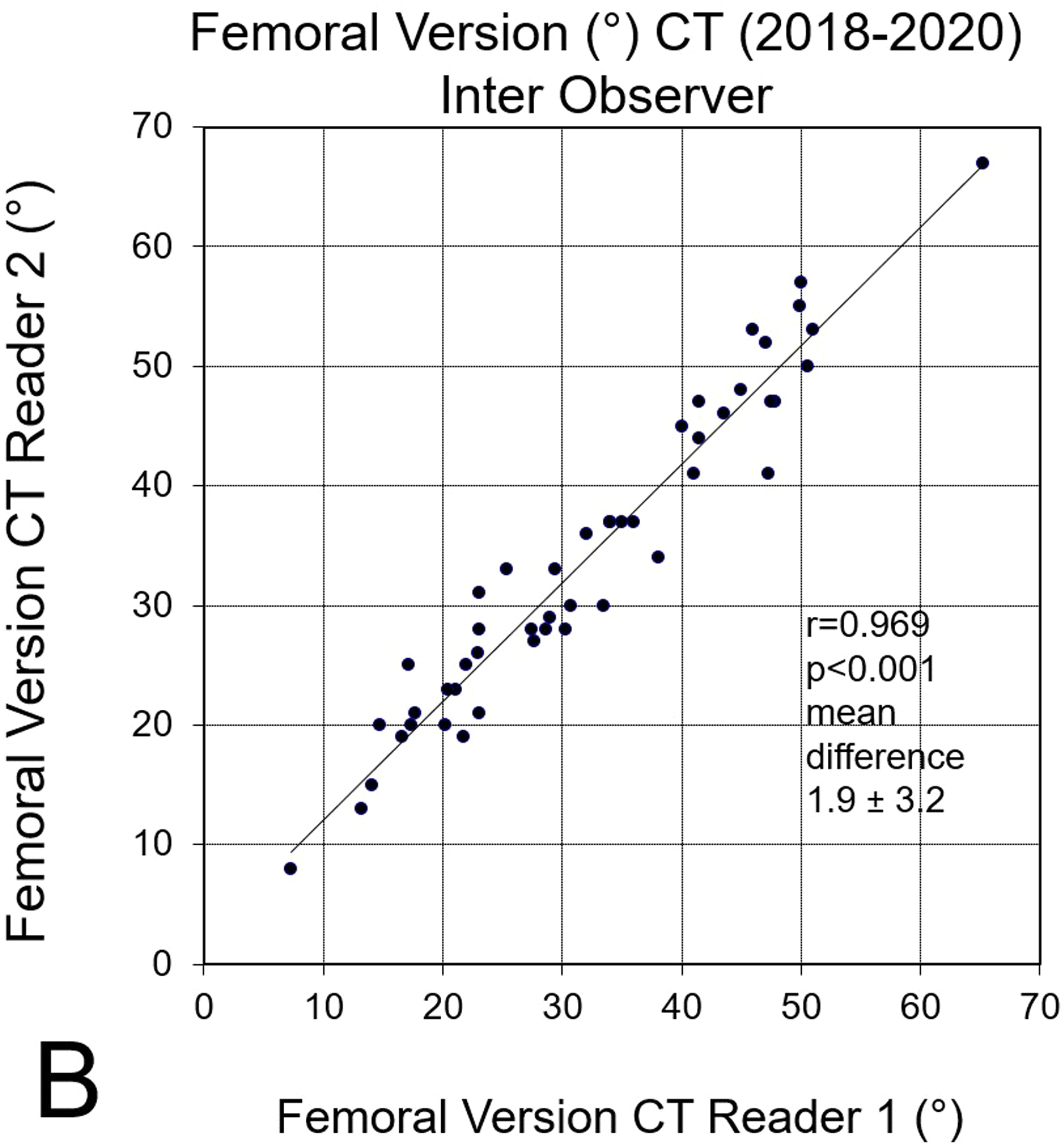

Supplement: Suppl Figure 3 — A-B Bland Altman analysis showing the difference between CT based and MRI based measurement of FV for reader 1 (A) and reader 2 (B). MRI was performed with fast Dixon based images [file mmc3.jpg]

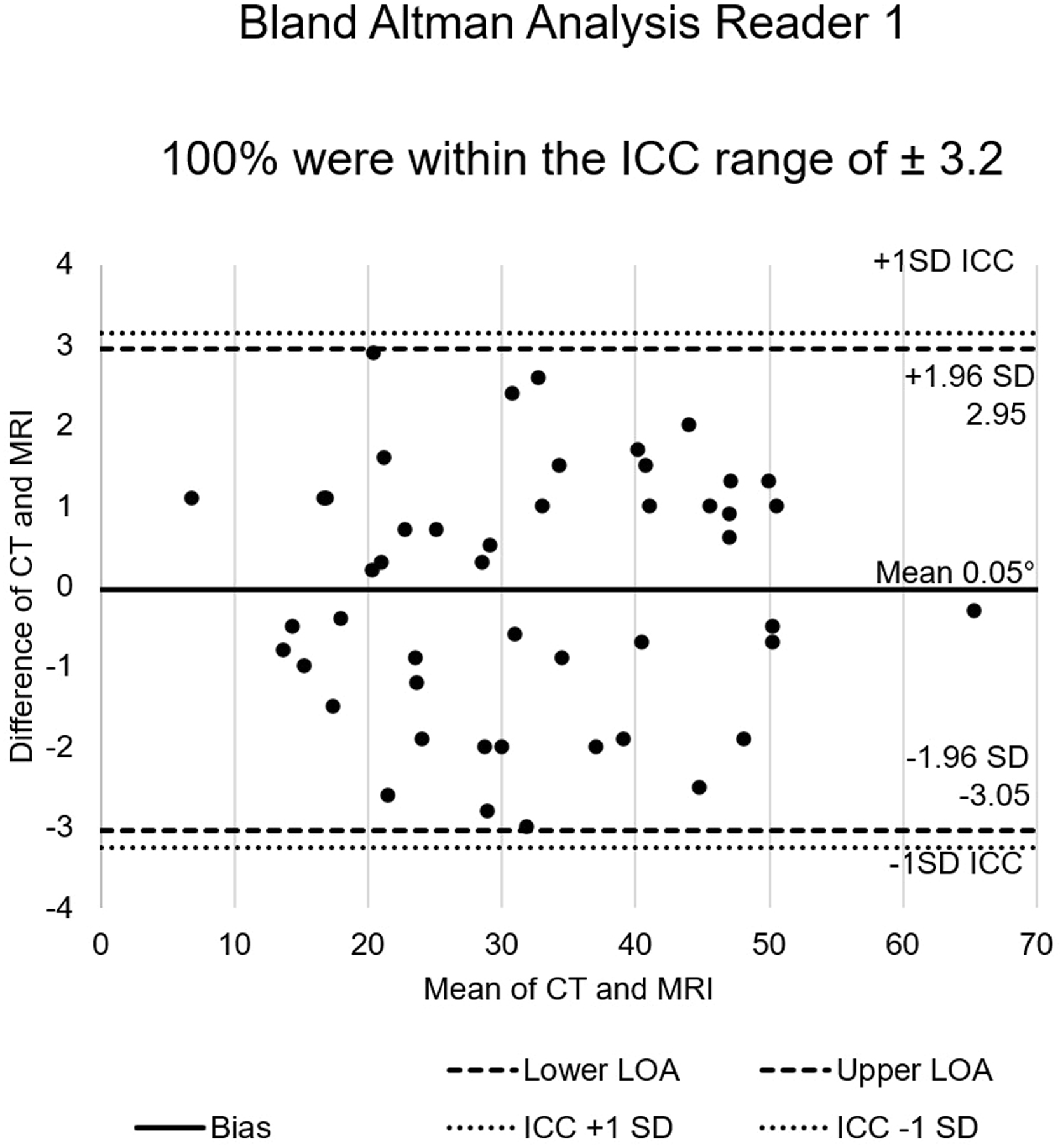

Supplement: Supplementary file 4 — Supplementary Material [file mmc4.jpg]

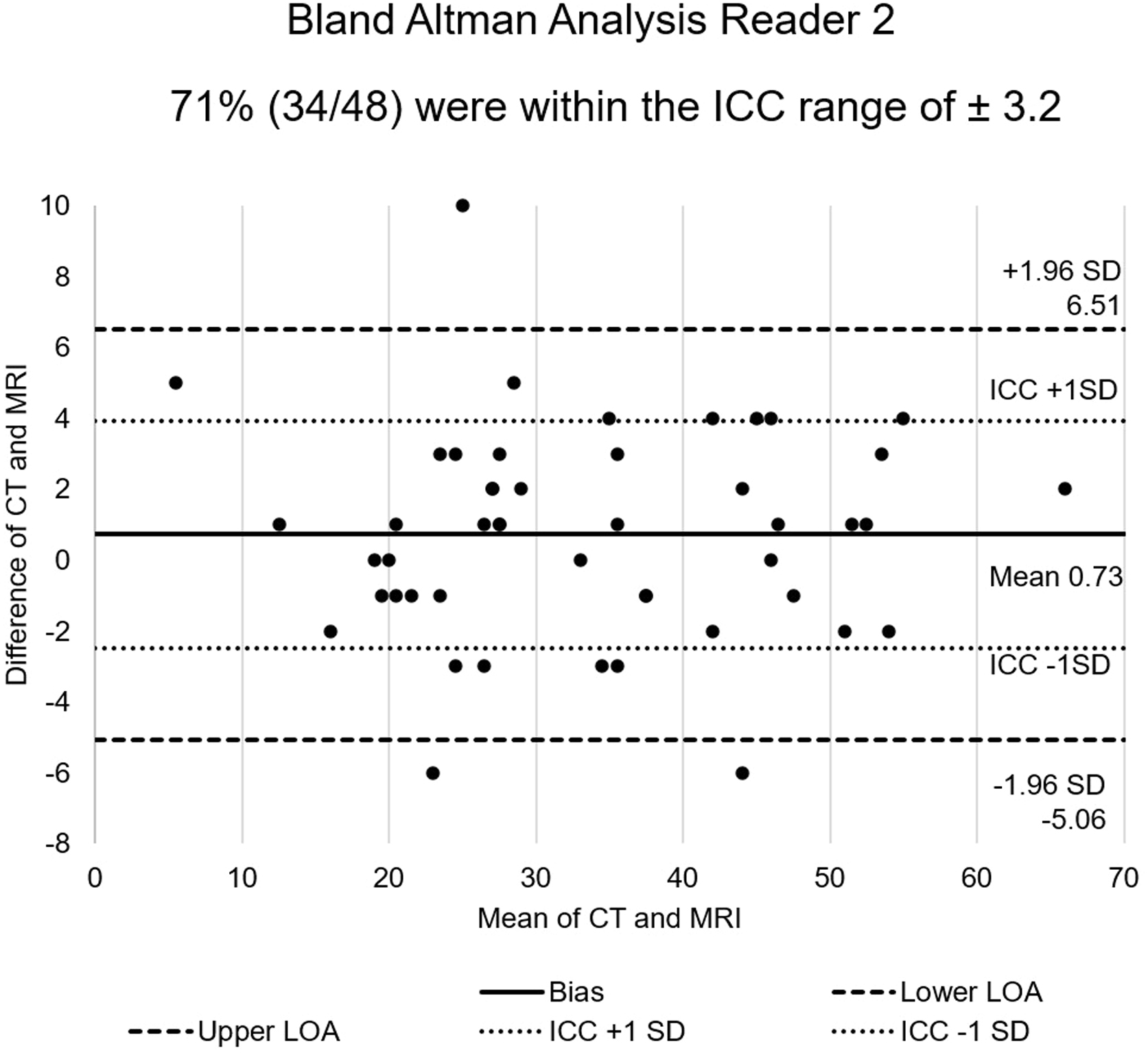

Supplement: Supplementary file 5 — Supplementary Material [file mmc5.jpg]
